# Draft Genome Sequences of Three Fusarium circinatum Isolates Used To Inoculate a Pedigreed Population of *Pinus elliottii* Seedlings

**DOI:** 10.1128/MRA.00631-20

**Published:** 2020-07-23

**Authors:** James C. Fulton, Jose C. Huguet-Tapia, Stephanie M. Adams, Nicholas S. Dufault, Tania Quesada, Jeremy T. Brawner

**Affiliations:** a Department of Plant Pathology, University of Florida, Gainesville, Florida, USA; b School of Forest Resources and Conservation, University of Florida, Gainesville, Florida, USA; c The Morton Arboretum, Lisle, Illinois, USA; Vanderbilt University

## Abstract

Here, we announce the draft genome sequences of three Fusarium circinatum isolates that were used to inoculate slash pines (Pinus elliottii) at the U.S. Forest Service Resistance Screening Center in Asheville, North Carolina. The genomes of these isolates were similar to other publicly available genomes, with average nucleotide identity values of >0.98.

## ANNOUNCEMENT

The fungus Fusarium circinatum (class Sordariomycetes, phylum Ascomycota) causes pitch canker disease in *Pinus* species (1, 2) and also infects Pseudotsuga menziesii (3, 4), maize, and native grasses (5). This diverse host range and its ability to infect seed have allowed the fungus to spread around the world (6, 7). Three isolates (S, V, and LB) collected from Florida, where pitch canker symptoms are prevalent (8), were selected from eight isolates previously used to inoculate southern pines (9).

The pathogens were isolated from pine trees with a spore trap and cultured for DNA extraction (10). Single-spore isolates were cultured on acidified quarter-strength potato dextrose agar (aqPDA) (BD Difco, Detroit, MI) and incubated at 25°C for 5 days. The leading edges of growing mycelia were extracted and ground with a micropestle in Eppendorf tubes with 200 ml of sterilized distilled water, and 50 μl of solution was streaked onto sterilized 100-mm cellophane disks (Bio-Rad Laboratories, Hercules, CA) laid on top of aqPDA, as performed by Cassago et al. (11). Cultures were grown for 48 h at 25°C to produce medium-free mycelia for storage in bead beater tubes, which were frozen in liquid nitrogen prior to lyophilization and homogenization with steel beads. DNA was extracted following an optimized phenol-chloroform genomic DNA extraction protocol described by Feehan et al. (12).

Samples were sequenced at the University of Florida Interdisciplinary Center for Biotechnology Research. Fifty nanograms of size-selected DNA was used for Illumina library preparation. Sequencing libraries were prepared using a NEBNext Ultra II DNA library preparation kit and NEBNext multiplex oligomers (New England Biolabs, Ipswich, MA) following the manufacturer’s protocol. Library enrichment and barcoding were performed with five to seven cycles of amplification prior to AMPure bead purification. The final library was quantitated with a Qubit 3.0 fluorometer (Thermo Fisher Scientific, Waltham, MA) and sized on the 2200 TapeStation (Agilent Technologies, Santa Clara, CA). DNA sequencing libraries were normalized and pooled equimolarly for sequencing in a single Illumina MiSeq v.3 run (2 × 300 cycles). Approximately 50 million high-quality paired-end reads (∼90% of reads had a score of at least Q30) yielded approximately 15 Gb of sequence data. Trim Galore v.0.6.5 was used to trim and pair raw reads, which were assembled into contigs using SPAdes v.3.13.0 with k-mer values of 21, 33, 55, 77, 99, and 127 (13, 14). Bowtie 2 v.2.3.5 aligned reads against the filtered contigs to produce the SAM format alignment (15). SAMtools v.1.10 converted these alignments to BAM files for polishing with Pilon v.1.22 using default parameters to output FASTA files (16, 17). Default parameters were used except where otherwise noted.

Table 1 summarizes assembly data and provides comparisons between publicly available references and newly sequenced isolates. The genomes of these isolates are similar to publicly available genomes from the American clade of the *Fusarium fujikuroi* species complex (18–21). Sequences include the conditionally disposable chromosome.

**TABLE 1:** General statistics and accession numbers for sequenced Fusarium circinatum genomes

Isolate	Genome size (bp)	No. of contigs	*N*_50_ (bp)	Avg coverage (×)	G+C content (%)	GenBank accession no.	ANI with^*a*^ :		
							FSP_34	KS17	GL1327
S	46,089,449	253	1,109,711	21	46.92	JABACQ000000000	0.993	0.982	0.994
LB	45,449,917	144	1,025,291	20	46.83	JABAYB000000000	0.993	0.982	0.994
V	46,349,683	248	853,138	19	46.94	JABACP000000000	0.992	0.981	0.993

a Average nucleotide identity (ANI) comparisons of *de novo*-sequenced Fusarium circinatum genomes with the publicly available F. circinatum assemblies of strains FSP_34, KS17, and GL1327 (GenBank accession numbers GCA_000497325.3, GCA_002894005.1, and GCA_000876485.1, respectively).

### Data availability.

Genome assemblies are available in GenBank under accession number PRJNA623862 for assemblies GCA_013168835.1, GCA_013168815.1, and GCA_013168825.1, while Illumina reads used for assembly are available under accession numbers SRX8085557, SRX8085558, and SRX8085556 for isolates S, V, and LB, respectively.
